# Virtual Reality-Incorporated Horse Riding Simulator to Improve Motor Function and Balance in Children with Cerebral Palsy: A Pilot Study

**DOI:** 10.3390/s21196394

**Published:** 2021-09-24

**Authors:** Hyun Jung Chang, Yong Gi Jung, Young Sook Park, Se Hwi O, Da Hye Kim, Chang Woo Kim

**Affiliations:** 1 Department of Physical Medicine and Rehabilitation, Samsung Changwon Hospital, Sungkyunkwan University School of Medicine, Changwon 51353, Korea; reh.chj@gmail.com (H.J.C.); jijibaeheiwon@daum.net (Y.S.P.); oskdoh@naver.com (S.H.O.); dahae2005@naver.com (D.H.K.); 2 Department of Otorhinolaryngology-Head and Neck Surgery, Samsung Medical Center, Sungkyunkwan University School of Medicine, Seoul 06351, Korea; ent.jyg@gmail.com

**Keywords:** horse riding simulator, virtual reality, motor function, balance, pediatrics, cerebral palsy

## Abstract

The horse riding simulator (HRS) reportedly has a beneficial effect on motor function and balance in children with cerebral palsy (CP). However, by itself, the HRS is not a sufficient source of challenge and motivation for children. To address this issue, we combined the HRS with virtual reality (VR) to promote somatosensory stimulation and motivation. Sixteen children (ages: 5–17 years) with CP and presenting Gross Motor Function Classification System (GMFCS) levels I–IV were enrolled in the study. Using a head-mounted display and controllers, interventions were carried out over 30-min periods (two rides lasting 12 min each, along with a six-min rest period) twice a week over a period of eight weeks (16 sessions in aggregate). The Pediatric Balance Scale (PBS), Gross Motor Function measure (GMFM)-88, and GMFM-66 scores of each participant were measured before and after the interventions. Statistically significant improvements were observed in the PBS, GMFM-66, the total GMFM-88 scores, and those corresponding to dimensions D and E of GMFM-88 after the intervention (*p* < 0.05). This study demonstrates that VR-incorporated HRS is effective in improving motor function and balance in children with CP and that its incorporation in conventional PT programs could yield beneficial results.

## 1. Introduction

Cerebral palsy (CP) refers to a group of permanent motor disorders attributed to a non-progressive lesion caused by an insult to or an anomaly in a developing brain [1]. In patients suffering from CP, the inability to control and coordinate voluntary muscles results in poor selective control of muscle activity. In addition, muscle contracture and/or alignment deformities in children degrade functional activity and make essential daily activities like balance control and walking difficult [2]. The management of people with CP is based on a framework that considers effective intervention programs as those that promote optimum function throughout the life span. A wide range of therapeutic and rehabilitation interventions are currently utilized to improve motor performance, motility, and independence in daily living [3].

Hippotherapy is a treatment strategy that uses equine movement as part of an integrated intervention program for achieving functional outcomes [4]. Several studies have presented evidence establishing the therapeutic efficacy of hippotherapy in children with CP; consequently, hippotherapy is suggested as a rehabilitative methodology for children with CP. During riding, multiple sensory inputs and efferent motor outputs stimulate the central nervous system, eventually leading to an improvement in postural stability and body alignment [5,6,7]. In particular, in clinical environments where real horses are not readily accessible or affordable, the horse riding simulator (HRS) has been used as an alternative. HRS imitates the passive movement consequent to horse riding and offers the additional advantage of enabling regular therapy with no apparent spatiotemporal or weather-related constraints. The HRS system considered in this study is designed to facilitate rhythmic trunk rotation, strength, stretching, and core stabilization based on the sensorimotor system.

Several studies have reported that HRS training improves static and dynamic balance abilities, postural control, and motor functions [8,9,10]. However, Lee et al. [8] compared hippotherapy and HRS therapy in terms of their effect of the static and dynamic balance in children with CP. They reported that there were balance improvements in both groups, but that a greater improvement was observed in the hippotherapy group. Further, Temcharoensuk et al. [11] compared the GMFM sitting ability score between hippotherapy and HRS therapy groups and reported that more improvement was observed in the hippotherapy group.

Ideally, pediatric neurorehabilitation should be fun, motivating, focused, and repetitive, as such exercises are meant to be performed over long periods to produce noticeable improvements in muscle size, motor behavior, and neuroplasticity [12]. However, the HRS does not provide any directional challenge, and it poses a constant postural challenge to the rider over the entire riding session. Therefore, children are likely to become bored with it after a certain period. This issue can be addressed by incorporating virtual reality (VR) training into the exercise, which is a therapeutic method that has been proven to engage children’s interests continuously. In particular, the combination of VR with a commercial game appeals to children and motivates them to acquire skills while playing the game in a safe environment [13]. Saposnik et al. [14] reported that VR training increased subjects’ interest in the exercise, thereby increasing their motivation for motor learning. Cho et al. [15] reported that treadmill training with VR was more effective than treadmill training without VR in enhancing functional activities.

The primary aim of this study was to evaluate the impact of a new training protocol, combining the HRS with VR training, on motor function and balance in children with CP.

## 2. Materials and Methods

### 2.1. Participants

This study was approved by the Institutional Review Board of Samsung Changwon Hospital (SCMC 2019-01-004). Signed informed consent forms were obtained from the parents of all participants and consent was also directly obtained from those participants older than 12 years of age after providing them with a detailed explanation of the study, including the risks and benefits.

The following inclusion criteria were used to select the participants: (1) positive CP diagnosis; (2) belonging to school-going or adolescent age groups; (3) positive diagnosis of Gross Motor Function Classification System (GMFCS) levels I–IV; and (4) the ability to sit upright on a static surface. The following exclusion criteria were adopted: (1) botulinum toxin injection in the previous three months; (2) orthopedic surgery in the previous six months; (3) selective dorsal rhizotomy operation; (4) HRS training in the previous six months; (5) difficulty in maintaining a sitting posture on HRS owing to pain or joint contracture; (6) uncontrolled epileptic seizure; (7) moderate to severe intellectual disability; and (8) poor visual or hearing acuity.

### 2.2. Interventions

**Figure 1:**
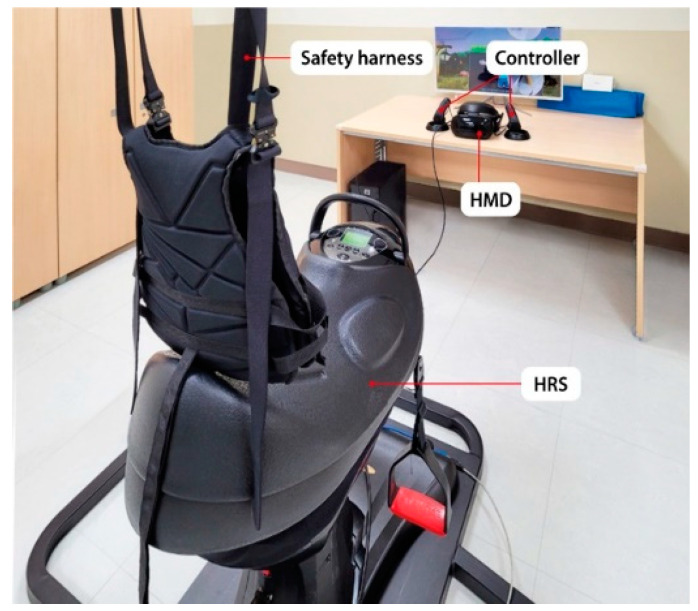
Architecture of the proposed virtual reality (VR)-incorporated horse riding simulator (HRS).

The participants were instructed to exercise using the HRS (Shin-Hwa EQ-900m, Ansan-si, Korea) equipped with a head-mounted display (HMD) and a pair of controllers (Samsung HMD odyssey, Seoul, Korea). The HRS system utilized in this study simulates the movement of a real horse. It is equipped with eight built-in exercise modes and is designed to implement an eight-shaped movement, which enabled the children to perform three-dimensional (3D) movements (back-and-forth, side-to-side, and up-and-down) like those on a real horse (Figure 1).

In total, it runs through six speed levels, with each level comprising two speed increments of 4 km/h each (e.g., level 1 begins at a speed of 14 km/h and lasts for 1 min, then the speed increases to 18 km/h and lasts for 4 min. Then, the speed increases to 22 km/h and lasts for 6 min). With each progressive level, the initial speed is increased by 4 km/h—level 1 begins at 14 km/h and Level 6 begins at 34 km/h. Based on the children’s proficiency in the HRS and progress in their postural core stability, the speed level was adjusted by a physician.

**Figure 2:**
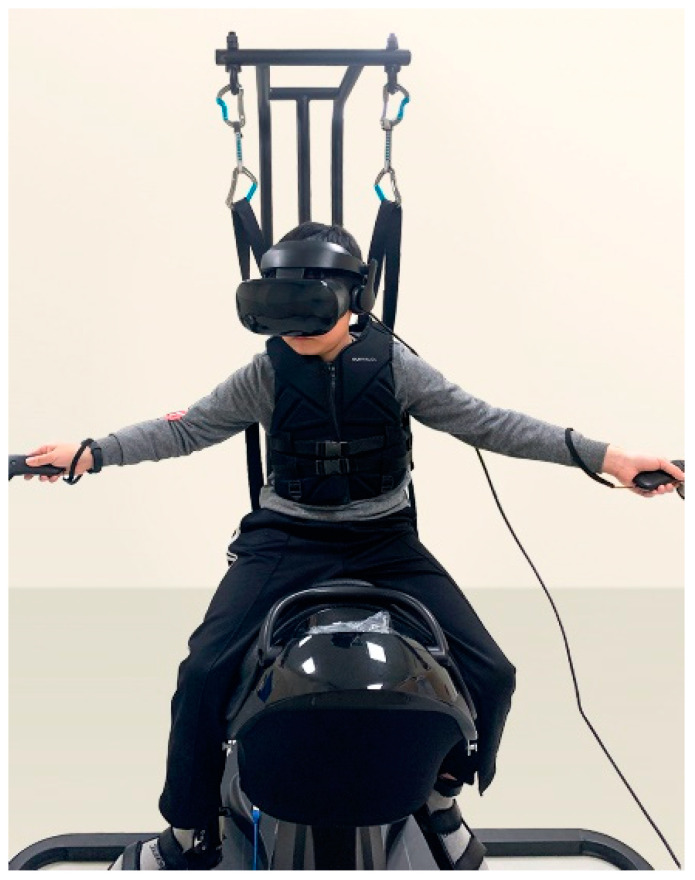
Children extend their hands to hit the target on the moving HRS.

Before initiating HRS training, the children were instructed to abduct their shoulders at 90° and 180° with their elbows fully extended with the controllers in their hands to allow them to settle into their own comfortable posture before beginning the intervention and calibration. The reaching distance of each child’s arms was measured by the HRS software to determine the location of the target in the VR program, and the postural challenge was maximized correspondingly. During the exercise, the children were instructed to extend their hands while holding the controller to hit a target on the moving HRS (Figure 2). In addition, they were required to tilt their trunk laterally on the HRS to avoid approaching obstacles.

To mitigate the danger of falling during the exercise, each participant was harnessed. Each intervention was carried out over a period of 30 min (12 min × 2 rides, along with a 6-min resting period) twice a week over a period of eight weeks, amounting to a total of 16 sessions. The blood pressure and heart rate of the participants were recorded before and after each intervention for safety reasons. After the entire intervention, a simple satisfaction survey was conducted with the participants and their parents. The questionnaire comprised the following questions: “Did you (or your child) have fun?” and “Do you (or your child) want to do it again?”

### 2.3. Outcome Measures

In this study, the Pediatric Balance Scale (PBS), Gross Motor Function Measure (GMFM)-88, and GMFM-66 scores of each participant were measured before the first intervention and after the completion of the eight-week program.

PBS, which is a modification of the Berg Balance Scale, was used to assess the participants’ balance. It consists of 14 items that are scored on a grading scale ranging from “0” to “4”. The maximum score possible is 56, with higher scores corresponding to better balance control. It evaluates the functional balance of children in the context of everyday tasks. The 14 items that constitute the PBS assess the following functional activities that are required to be performed by children safely and independently at home, school, or in the community: sitting balance, standing balance, transitioning from sitting to standing position, transitioning from standing to sitting position, transferring, stepping, reaching forward, reaching to the floor, turning, and stepping onto and off an elevated surface. It has been reported to exhibit good reliability in the case of children of school age with mild to moderate motor impairment [16].

GMFM and its subsequent revision have become the most common functional outcome measure used by rehabilitation specialists to measure gross motor functioning in children with CP and other neurologically-based conditions [17].

GMFM-88 has been demonstrated to exhibit high validity, reliability, and responsiveness in evaluating motor function and assessing the results of management strategies on children with CP [16]. In this study, GMFM-88 was used to assess clinical changes in the participants. It was organized in terms of five separate dimensions: (A) lying and rolling; (B) sitting; (C) crawling and kneeling; (D) standing; and (E) walking, running, and jumping. The levels of each item were explicitly defined and scored on a scale of 0–3. the individual item scores were summed to yield the scores corresponding to each dimension.

GMFM-66 was developed using Rasch analysis in an attempt to improve the interpretability and clinical usefulness of the earlier measure, with 22 of the original 99 items deleted to improve reliability and validity. The 22 deleted items are comprised of 13 from the lying and rolling dimension, five from the sitting dimension, and four from the kneeling and crawling dimension [18]. GMFM-66 provides detailed information on the level of difficulty of each item, thereby providing more information to assist with goal setting. Thus, it is recommended for research purposes when comparing changes in gross motor function over time in children with CP [19]. However, GMFM-66 is less useful when scoring children with a severe disability [3]. Therefore, both GMFM-66 and GMFM-88 were used to assess the gross motor function of children with CP in this study.

The items were administered in the same manner for both GMFM-66 and GMFM-88. The GMFM-66 scores were calculated from GMFM-88 using the Gross Motor Ability Estimator. The GMFM and PBS scores of each participant were evaluated before and after the entire intervention by the same examiner.

### 2.4. Statistical Analysis

SPSS ver. 21.0 software (IBM, Inc., Chicago, IL, USA) was used for statistical analysis. The Wilcoxon signed-rank test was used to compare the values of the outcome measures before and after the intervention. A *p*-value of 0.05 was considered to be statistically significant for each analysis.

## 3. Results

A total of 16 children (ages: 5–17 years) with CP exhibiting variable motor functions (GMFCS levels I–IV) were enrolled as participants in the study. The participants comprised six females and ten males. The number of children for each GMFCS level was as follows: level I (n = 6); level II (n = 6); level III (n = 1); level IV (n = 3). The demographic characteristics and clinical data of the children are listed in Table 1.

On average, statistically significant improvements in the PBS score were observed, with its value increasing from 35.81 ± 15.68 before the interventions to 40.63 ± 13.07 after the interventions. Moreover, statistically significant improvements in the GMFM-66 and GMFM-88 total score and those corresponding to dimensions D and E were also observed, with GMFM-66 score increasing from 69.26 ± 17.65 before the interventions to 70.65 ± 18.47 after the interventions and the GMFM-88 total score increasing from 81.20 ± 21.54 before the interventions to 82.33 ± 20.28 after the interventions. Among the GMFM-88 scores, the dimension D score increased from 71.63 ± 30.80 before the interventions to 73.56 ± 30.60 after the interventions, and the dimension E score increased from 60.59 ± 34.84 before the interventions to 61.29 ± 34.78 after the interventions. However, no significant difference was observed corresponding to dimensions, A, B, and C, of GMFM-88 (Table 2).

The participants did not suffer from any adverse events, such as falls, pain, dizziness, or sudden changes in blood pressure and heart rate. Following the completion of the interventions, the children expressed interest and enjoyment in the exercise, and the feedback of their parents regarding VR-incorporated HRS was equally positive.

## 4. Discussion

The primary purpose of this study was to investigate the effect of VR-incorporated HRS training on motor function and balance in children with CP. The results of the study revealed that the intervention improved both the GMFM scores (both GMFM-66 and GMFM-88) and the PBS scores. A key strength of this work is that it is a first attempt to conduct a study on 3D, VR-incorporated, HRS training to motivate children with adequate challenges to arm posture and body sway. The GMFM is the most widely used measure in the evaluation of CP. The technique used in this study was demonstrated to result in improvements in the GMFM-66 and GMFM-88 total scores, particularly with enhancements in the aspects corresponding to dimensions D and E of the GMFM-88 score.

Several previous studies have reported HRS training to be a positive intervention method for improvement in gross motor function. Park et al. [20] reported that the postural muscle size and postural stability in the static and dynamic states were improved after the administration of robotic hippotherapy once per week for 12 weeks in a child with CP. Kim et al. [21] reported significant improvements in gait velocity, sway velocity and sway distance in five children with CP following HRS training twice per week over a period of 4 weeks. In addition, Hemachithra et al. [22] reported HRS to be successful in reducing abductor spasticity and improving the range of hip abduction in 12 children. A reduction in spasticity can lead to an improvement of gross motor function in children with CP. In this regard, Valevski et al. [23] reported that a decrease in the spasticity of lower extremity muscles, resulting from injections of botulinum toxin A, led to the improvement of GMFM scores in patients with CP.

The GMFM score quantifies a child’s proficiency in performing complex patterns of movement, including trunk balance and coordination, as well as its strength and mobility. Although the exact mechanism by which HRS improves gross motor function in children with CP is not known, it may facilitate upright posture, equilibrium reaction, stretching of shortened hip abductors, and strengthening of the lumbopelvic musculature [20]. The ability to maintain various standing positions and perform specific tasks from the standing position is also improved owing to the increase in the stability of the base of support [24]. These improvements in balance control yield a positive influence on the GMFM score, especially dimensions D and E. Gan et al. [25] and Ko et al. [26] previously reported that PBS was highly correlated with the GMFM total score, dimension D, dimension E, walking speed, and the 10 s sit to start test. The results of our study also demonstrate significantly greater improvement in the PBS, GMFM total, dimension D, dimension E, and GMFM-66 scores. The deletions from GMFM-88 to make GMFM-66 (13 items deleted from dimension A, five from dimension B, and four from dimension C) may have influenced the greater gain observed in GMFM-66. Dimensions A, B, and C of GMFM-88 were observed to be unresponsive in the children participating in this study. This can be attributed to the ceiling effect of GMFM. Since most of the children participating in this study (GMFCS levels I–IV) exhibited good sitting ability in order to undertake the HRS training, dimensions A, B, and C of GMFM were relatively easy for them.

The improvement in the PBS score noted in this study is consistent with the observations of previous studies that reported improvements in postural control following HRS training [9,10,27]. They reported that HRS training significantly improves the postural control in children with CP. Lee et al. [8] compared the therapeutic effects of hippotherapy with those of HRS and found that both techniques improved the PBS scores and decreased body sway length, with no significant difference observed between their effects. In contrast, Temcharoensuk et al. [11] compared hippotherapy with HRS therapy in terms of sitting stability in 30 children with bilateral spastic CP and reported that the hippotherapy group exhibited greater improvement than the HRS group. However, compared to the current study, their study used only HRS, and the participants were asked to hold a handle during HRS therapy, which may have reduced the postural challenge. In contrast, in this study, postural challenges such as extending the arms and tilting the trunk were performed without holding the handle. These activities could have affected their performance.

Ideally, the rehabilitation of postural control in children with balance deficits should include activities that address factors that constrain musculoskeletal, motor, and sensory processing speeds. In addition, such interventions should focus on static and dynamic equilibrium tasks during practice in order to actively engage the children [28,29]. HRS therapy is a task-oriented training that satisfies these requirements. Further, to optimize skill acquisition during task-oriented training, the training must be sufficiently challenging to facilitate learning and promote enterprising and adaptable perspectives [30]. This would motivate children to continue to acquire or modify new skills. However, the HRS does not produce changing patterns and directions similar to those experienced during real horseback riding. In other words, the HRS induces fewer signals from the proprioceptive and vestibular receptors, which are stimulated by various postural challenges. Thus, by itself, the HRS is insufficient to challenge and motivate children.

In the current study, most participants experienced HRS training in conjunction with VR therapy for the first time; thus, the set of tasks was novel to them, involving massive postural challenges and arousing sufficient interest. Further, the VR system equipped with the HMD provided children with a 3D scene, mimicking real horseback riding. Thus, the participants found VR-incorporated HRS training to be an exceedingly enjoyable and meaningful activity. The beneficial effect of VR-incorporated HRS training can also be potentially attributed to the increasingly difficult postural challenges provided by the VR system, by incorporating movements such as the stretching or lifting of arms as much as possible without holding the handle, or the tilting of the trunk while using the HRS. As the children could not hold onto a handle during the intervention, they were forced to activate their lower extremities, hip muscles, and trunk muscles to compensate for the unstable postural changes of the trunk on a continuous moving saddle. This strengthened the muscles in their lower extremity and exerted a positive influence on postural balance and gross motor function.

This study has several limitations that need to be noted while considering our findings; these drawbacks should be addressed and overcome in future studies. First, the sample size was small and heterogeneous. Sixteen children with GMFCS levels I–IV were included in this study. Kwon et al. [31] reported that the scores corresponding to the GMFM dimensions that benefited from the effect of therapy were dependent on the participants’ GMFCS levels. Therefore, further studies are required to evaluate the effect of VR-incorporated HRS training on each GMFCS dimension. Second, because this study did not control other therapeutic activities undertaken by the participants following an ethical principle, the isolated effect of VR-incorporated HRS on motor function could not be evaluated. Therefore, additional randomized controlled trials should be conducted to evaluate it. Third, the current study only investigated the short-term effects of VR-incorporated HRS training on children with CP; future studies must assess its sustained effects over time. Finally, this was a preliminary study to investigate the effect of VR-incorporated HRS in improving the motor function and balance in children with CP in which the results showed positive effects. As a pilot study for a future large-scale therapy-control group study, this study has limitations. The fact that there was a lack of sample diversity owing to the small and heterogeneous sample, in addition to absence of a control group, might have influenced the results and should not be neglected. Therefore, further randomized controlled trials involving large sizes should be conducted to assess the effect of VR and changes in HRS training on the motor function and balance in children with CP and other conditions.

## 5. Conclusions

This study analyzed the effect of VR-incorporated HRS training on motor function and balance in children with CP. Our results revealed statistically significant improvements in the PBS score, GMFM-66, GMFM-88 total score, and those corresponding to dimensions D (standing) and E (walking, running, and jumping) in GMFM-88, without any adverse effects. These findings suggest that the proposed VR-incorporated HRS methodology can be considered a viable therapeutic approach for improving motor function and balance ability in children with CP.

## Figures and Tables

**Table 1:** Demographic characteristics of the participants.

	Sex	Age (years)	Neuromotor Type	Height (cm)	Weight (kg)	GMFCS
Case 1	M	10	Spastic Unilateral	140.1	45.4	I
Case 2	M	7	Spastic Unilateral	122.1	22.2	I
Case 3	M	5	Spastic Unilateral	112.6	20.1	I
Case 4	M	12	Spastic Bilateral	154.6	56.0	I
Case 5	F	9	Spastic Unilateral	125.5	36.3	I
Case 6	M	11	Ataxic	154.9	68.7	I
Case 7	F	10	Spastic Bilateral	124.4	25.5	II
Case 8	M	13	Spastic Bilateral	145.5	58.6	II
Case 9	M	17	Spastic Bilateral	162.2	50.5	II
Case 10	M	8	Spastic Bilateral	107.8	19.0	II
Case 11	F	14	Spastic Bilateral	126.6	29.0	II
Case 12	F	10	Spastic Bilateral	121.4	23.5	II
Case 13	M	5	Spastic Bilateral	107.0	15.3	III
Case 14	F	13	Spastic Bilateral	136.0	41.0	IV
Case 15	F	6	Spastic Bilateral	103.0	17.3	IV
Case 16	M	11	Spastic Bilateral	136.6	40.0	IV
Mean ± SD	M: 10, F: 6	10.06 ± 0.84		130.64 ± 4.90	36.33 ± 4.46	

Values are presented as mean ± standard deviation. GMFCS, Gross motor function classification system.

**Table 2:** Changes in Pediatric Balance Scale and Gross Motor Function Measure scores.

	Preintervention	Postintervention	*p*-Value
PBS (%)	35.81 ± 15.68	40.63 ± 13.07	0.001 *
GMFM-66 (%)	69.26 ± 17.65	70.65 ± 18.47	0.006 *
GMFM-88 (%)			
A	94.98 ± 8.25	96.20 ± 5.43	0.285
B	93.23 ± 16.14	93.86 ± 14.22	0.251
C	85.57 ± 24.43	86.76 ± 22.32	0.178
D	71.63 ± 30.80	73.56 ± 30.59	0.001 *
E	60.58 ± 34.84	61.29 ± 34.78	0.037 *
Total	81.20 ± 21.54	82.33 ± 20.28	0.021 *

NOTE. Values expressed as mean percentage standard deviation. * Statistically significant difference between pre and post intervention (*p* < 0.05). PBS, Pediatric balance scale; GMFM, Gross Motor Function Measure; A, lying and rolling; B, sitting; C, crawling and kneeling; D, standing; E, walking, running, and jumping.
